# RECQL4 regulates DNA damage response and redox homeostasis in esophageal cancer

**DOI:** 10.20892/j.issn.2095-3941.2020.0105

**Published:** 2021-02-15

**Authors:** Guosheng Lyu, Peng Su, Xiaohe Hao, Shiming Chen, Shuai Ren, Zixiao Zhao, Yaoqin Gong, Qiao Liu, Changshun Shao

**Affiliations:** 1 Key Laboratory of Experimental Teratology, Ministry of Education, Department of Molecular Medicine and Genetics, School of Basic Medical Sciences, Shandong University, Jinan 250012, China; 2 Department of Pathology, Qilu Hospital of Shandong University, Jinan 250012, China; 3 State Key Laboratory of Radiation Medicine and Protection, Institutes for Translational Medicine, Soochow University, Suzhou 215123, China

**Keywords:** ESCC, RECQL4, senescence, redox, DNA damage response

## Abstract

**Objective:**

RECQL4 (a member of the RECQ helicase family) upregulation has been reported to be associated with tumor progression in several malignancies. However, whether RECQL4 sustains esophageal squamous cell carcinoma (ESCC) has not been elucidated. In this study, we determined the functional role for RECQL4 in ESCC progression.

**Methods:**

RECQL4 expression in clinical samples of ESCC was examined by immunohistochemistry. Cell proliferation, cellular senescence, the epithelial-mesenchymal transition (EMT), DNA damage, and reactive oxygen species in ESCC cell lines with RECQL4 depletion or overexpression were analyzed. The levels of proteins involved in the DNA damage response (DDR), cell cycle progression, survival, and the EMT were determined by Western blot analyses.

**Results:**

RECQL4 was highly expressed in tumor tissues when compared to adjacent non-tumor tissues in ESCC (*P* < 0.001) and positively correlated with poor differentiation (*P* = 0.011), enhanced invasion (*P* = 0.033), and metastasis (*P* = 0.048). RECQL4 was positively associated with proliferation and migration in ESCC cells. Depletion of RECQL4 also inhibited growth of tumor xenografts *in vivo*. RECQL4 depletion induced G0/G1 phase arrest and cellular senescence. Importantly, the levels of DNA damage and reactive oxygen species were increased when RECQL4 was depleted. DDR, as measured by the activation of ATM, ATR, CHK1, and CHK2, was impaired. RECQL4 was also shown to promote the activation of AKT, ERK, and NF-kB in ESCC cells.

**Conclusions:**

The results indicated that RECQL4 was highly expressed in ESCC and played critical roles in the regulation of DDR, redox homeostasis, and cell survival.

## Introduction

Esophageal squamous cell carcinoma (ESCC) is one of the common cancers worldwide, particularly in China, where ESCC ranks as the fourth most common cause of cancer-related death^1,2^. Although advances in diagnosis and treatment have improved clinical outcomes to some extent, the prognoses of patients with ESCC still remain poor, with a 5-year overall survival rate ranging from 20% to 30%^3,4^. Therefore, a better understanding of the molecular mechanisms underlying ESCC initiation and progression will facilitate the identification of novel early diagnostic markers and effective therapeutic strategies.

RECQL4, a member of the RECQ helicase family, has been shown to participate in many aspects of DNA metabolism^5–7^. Accumulated evidence indicates that RECQL4 plays a critical role in both nuclear and mitochondrial genome maintenance and stability, including the initiation of DNA replication, telomere maintenance, progression of stalled replication forks, and repair of DNA double-strand breaks (DSBs)^8–15^. Mutation of RECQL4 causes Rothmund-Thomson syndrome (OMIM 268400), an autosomal recessive disorder that is characterized by abnormalities in the skin and skeleton, juvenile cataracts, premature aging, and a predisposition to neoplasia^16^. Furthermore, somatic deletion of Recql4 leads to failure in hematopoiesis. Notably, ATP-dependent helicase activity is dispensable for the physiological function of RECQL4^17,18^. Recent studies have shown that RECQL4 overexpression occurs in prostate cancer, sporadic osteosarcoma, sporadic breast cancer, colorectal cancer, liver cancer, and gastric cancer, and is significantly associated with clinical outcomes^19–23^. Moreover, depletion of RECQL4 in cancer cells has been shown to significantly reduce cell proliferation and cell invasion potential, promote apoptosis, and impair tumorgenicity in tumor-bearing mice^20,24,25^. RECQL4 has also been shown to be associated with cisplatin resistance in gastric cancer by activating AKT^26^. However, whether and how RECQL4 may function in sustaining the malignancy of ESCC has not been elucidated.

In this study, we examined RECQL4 expression in ESCC tissues and evaluated the association between RECQL4 expression and clinicopathological features in ESCC patients. We found that RECQL4 expression was significantly increased in human ESCC tissues, especially in metastatic tissues. We then established stable RECQL4-knockdown ESCC cell lines (KYSE30 and TE-1) and RECQL4-overexpression ESCC cell lines (KYSE150 and KYSE410) and analyzed cell proliferation, cell cycle distribution, the levels of DNA damage and reactive oxygen species, cell migration, and invasion. We observed that RECQL4 was required for cell proliferation and migration of ESCC. RECQL4 depletion induced reactive oxygen species and DNA damage in ESCC cells, which was accompanied by cell cycle arrest and cell senescence. In addition, DNA damage response, as reflected by the phosphorylation of ATM and other kinases, was impaired when RECQL4 was depleted. RECQL4 was also found to promote the activation of AKT, ERK, and NF-kB in ESCC cells. Collectively, our results showed that RECQL4 was involved in the regulation of the DNA damage response, redox homeostasis, and cell survival in ESCC.

## Materials and methods

### Tissue specimens

ESCC specimens were collected from 94 patients between January 1, 2014 and July 30, 2018 in the Department of Pathology, Qilu Hospital of Shandong University. The use of the specimens was approved by the Ethics Committee of Shandong University School of Medicine (Approval No. ECSBMSSDU2019-1-052). A tissue microarray (TMA), containing 103 ESCC tissues and their corresponding normal esophageal tissues, was purchased from Shanghai Outdo Biotech Co., Ltd. (Shanghai, China). Follow-up information was obtained by reviewing patient medical records.

### Immunohistochemical staining

Immunohistochemical (IHC) staining was performed using a PV-9000 IHC Kit (Zhongshan Goldenbridge Biotechnology, Beijing, China). Briefly, paraffin sections of human ESCC samples were heated, dewaxed, and hydrated in xylene and ethanol/H_2_O. Antigens were retrieved by microwave oven heating (10 min) at middle power setting in EDTA buffer (pH 9.0). The sections were then incubated in 3% hydrogen peroxide in absolute methanol at room temperature for 10 min to block endogenous peroxidase activity. After 3 rinses (each for 5 min) in phosphate-buffered saline (PBS), the sections were incubated with rabbit anti-human RECQL4 antibody (1:100, Proteintech, Rosemont, IL, USA), diluted in phosphate-buffered saline (PBS) overnight at 4 °C, with 150 μL polymerized horseradish peroxidase-conjugated anti-rabbit IgG for 30 min at room temperature. The reaction products were visualized with diaminobenzidine (DAB Kit; Zhongshan Goldenbridge Biotechnology) and the slides were counterstained with hematoxylin, dehydrated, and evaluated using a light microscope. Negative controls omitted the treatment with primary antibodies. The protein expression level of RECQL4 was then evaluated by microscopic examination of the stained tissue slides. RECQL4 expression levels were determined using a visual immunoreactive score (IRS), which was generated by staining intensity (SI) × number of stained cells. The SI was scored as follows: negative (score: 0), weak (score: 1), moderate (score: 2), and strong (score: 3). We scored the positively or negatively stained tumor cells in the field as: score: 0 (negative), score: 1 (1%–25%), score: 2 (26%–50%), score: 3 (51%–75%), and score: 4 (> 76%). If the IRS score was > 8, the expression of RECQL4 was defined as high, if the IRS score was 5–8, the expression of RECQL4 was defined as medium, and an IRS score of ≤ 4 was defined as low or none.

### Cell lines

The human ESCC lines, KYSE30, KYSE450, KYSE410, KYSE150, and TE-1, were obtained from the Cancer Institute and Hospital, Chinese Academy of Medical Sciences (Beijing, China) and cultured in RPMI 1640 supplemented with 10% fetal bovine serum, 100 units/mL penicillin, and 100 mg/mL streptomycin. KYSE30, KYSE450, KYSE410, and KYSE150 cells were originally generated by Dr. Yutaka Shimada. All cells were grown at 37 °C in a humidified incubator containing 5% CO_2_.

### The cDNA synthesis and real-time PCR

RNA was isolated using TRIzol reagent (Invitrogen, Carlsbad, CA, USA) according to the manufacturer’s protocol. The cDNA was synthesized by reverse transcription of 1 μg of total RNA with random hexamers. The total volume of the reverse transcription reaction was 20 μL. Real-time quantitative reverse transcription PCR was performed using the LightCycler 480 sequence Detection System (Roche Applied Science, Basel, Switzerland). The average threshold cycle (Ct) of quadruplicate reactions was determined, and amplification was analyzed by the ΔΔCt method. Human glyceraldehyde 3-phosphate dehydrogenase (GAPDH) was amplified as an internal control. The levels of RECQL4 and GAPDH mRNA were measured by the SYBR Green I (Roche Diagnostics GmbH, Mannheim, Germany) assay. RECQL4 was amplified by using the primers with sequences 5′-GCGCTCTACCGGGAATACC-3′ (forward) and 5′-CAGCCCGATTCAGATGGGG-3′ (reverse). The GAPDH primers were 5′-CAGAACATCATCCCTGCCTCTAC-3′ (forward) and 5′-TTGAAGTCAGAGGAGACCACCTG-3′ (reverse). The samples were loaded in quadruple, and the results of each sample were normalized to GAPDH.

### Western blot analysis

The cells were harvested after treatment, rinsed in ice-cold PBS, and lysed in lysis buffer containing 50 mmol/L HEPES (pH 7.9), 0.4 mol/L NaCl, 1 mmol/L EDTA, 2 mg/mL leupeptin, 2 mg/mL aprotinin, 5 mg/mL benzamidine, 0.5 mmol/L phenylmethylsulfonylfluoride, and 1% NP40. Equal amounts of protein were separated by 10% SDS-PAGE, and transferred to polyvinylidene difluoride membranes (Millipore, Burlington, MA, USA), and blocked with 5% nonfat dry milk in TBS-Tween-20 (0.1%, v/v) for 1 h at room temperature. The membrane was incubated with primary antibody overnight. Antibodies to RECQL4 (1:1,000), p-ATM (1:1,000), ATM (1:1,000), p-ATR (1:1,000), ATR (1:1,000), p-CHK1 (1:1,000), CHK1 (1:1,000), p-CHK2 (1:1,000), CHK2 (1:1,000), γ-H2AX (1:1,000), p-p65 (1:1,000), P65 (1:1,000), p-ERK1/2 (1:1,000), ERK1/2 (1:1,000), p-AKT (1:1,000), and AKT (1:1,000) were purchased from Cell Signaling Technology (Danvers, MA, USA); anti-E-cadherin (1:1,000), anti-BAX (1:1,000), anti-Bcl-2 (1:1,000), anti-p21 (1:1,000), and anti-GAPDH (1:10,000) were from Proteintech (Wuhan, China); anti-CHK1 (1:200) was from Santa Cruz Biotechnology (Santa Cruz, CA, USA); and anti-vimentin (1:500), anti-cyclin D (1:500), anti-cyclin E (1:500), and anti-c-myc (1:500) were from Affinity Biosciences (Changzhou, Jiangsu, China). After washing, the membrane was incubated with the appropriate horseradish peroxidase-conjugated secondary antibody (diluted 1:5,000; Amersham Pharmacia Biotech, Little Chalfont, UK) for 1 h. After several washes, the blots were visualized using enhanced chemiluminescence (Thermo Fisher Scientific, Waltham, MA, USA).

### Small interfering RNA (siRNA) transfection

RECQL4 siRNA duplex 1, 2, and 3 (cat. nos. SASI_Hs01_00135884, SASI_Hs01_00135885, and SASI_Hs02_000337217) and UNIV NEGATIVE CONTROL small interfering RNA (siRNA; #2) were synthesized (Sigma-Aldrich, St. Louis, MO, USA) and transfected into KYSE30 and TE-1 cells using Lipofectamine 2000 (Invitrogen) for 24 h. RECQL4 protein levels were determined by Western blot analysis and the cell cycle was examined as described above.

### Lentivirus-mediated shRNA infection

The RECQL4 shRNA was in the tet-on inducible shRNA lentiviral vector [LV3 (H1/GFP&Puro)] and was purchased from Shanghai GenePharma (Shanghai, China). The target sequence was 5′-CCTAGACAGAGGGAACTATAT-3′ as previously described^27^ and verified by sequencing. Lentiviral particle production and infection were performed as described previously^28^. KYSE30 and TE-1 cells were infected by lentiviral particles in 6-well plates, followed by puromycin selection (Sigma-Aldrich) for approximately 2 weeks to select stably-expressing cells.

### Plasmid transfection

KYSE150 and KYSE410 cells were transfected with the pcDNA3.1-RECQL4 overexpression plasmid and the pcDNA3.1 control empty plasmid, using Lipofectamine 2000 according to the manufacturer’s instructions. Transfected cells were maintained in appropriate growth medium supplemented with 600 μg/mL G418 for 14 days to select neomycin-resistant colonies.

### Colony formation assay

Cells were trypsinized, counted, and plated into 6-well plates and cultured for 10–14 days. At the end-point, the cells were washed, fixed with methanol and stained with Crystal Violet. The number of colonies containing at least 50 cells in 10 random view fields was counted using a microscope.

### EdU incorporation

EdU (Cell-Light EdU Cell Proliferation Detection kit; Guangzhou RiboBio, Guangzhou, China) was added at 50 μmol/L and the cells were cultured for an additional 1 h. After the removal of EdU-containing medium, the cells were fixed with 4% paraformaldehyde at room temperature for 30 min, washed with glycine (2 mg/mL) for 5 min in a shaker, treated with 0.2% Trion X-100 for 10 min, and washed with PBS twice. Click Reaction Buffer (100 mmol/L Tris-HCl, pH 8.5, 1 mmol/L CuSO_4_, 100 μmol/L Apollo 550 fluorescent azide, and 100 mmol/L ascorbic acid) was then added. After 10–30 min, the cells were washed with 0.5% Triton X-100, 3 times, stained with 4′,6-diamidino-2-phenylindole (DAPI) for 10 min at room temperature, washed with 0.5% Triton X-100 for 5 times, and, finally immersed in 150 μL of PBS and examined using a fluorescence microscope.

### Analysis of cell cycle and apoptosis by flow cytometry

Cells were harvested using 0.25% trypsin-EDTA, centrifuged (300 × *g*), and washed once with cold PBS. The pellet was resuspended in ice-cold 70% ethanol and stored at –20 °C. The samples were incubated with 20 μg/mL propidium iodide/0.1% Triton X-100 staining solution with 0.1 mg/mL RNase A. Cell-cycle distribution was determined using the BD Biosciences FACS Canto II Analyzer (BD Biosciences, San Jose, CA, USA). At least 20,000 cells were collected. For analysis of apoptosis, both adherent and floating cells were harvested, washed twice in PBS, and resuspended in 1× binding buffer at a density of 1 × 10^6^ cells/mL. The cells were assayed for apoptosis using a PE-Annexin V Apoptosis Detection Kit I (BD Pharmingen, San Jose, CA, USA) according to the manufacturer’s instructions. For these studies, all experiments were repeated 3 or more times.

### Senescence-associated acidic ***β***-galactosidase staining

A senescence β-Galactosidase Staining Kit purchased from Cell Signaling Technology was used. Four high power fields per sample were counted in 3 independent samples to score the number of senescent cells.

### Wound healing and cell migration assays

Cell migration was assessed using a wound healing assay and Transwell assay. Briefly, for the wound healing assay, the cells were cultured in 6-well plates with serum-free culture medium. A scratch lesion was created using a 200 μL pipette tip. Then, the culture medium was removed and the wells were washed gently with PBS to remove dislodged cells. The wound was immediately photographed and at 12 h and 24 h after scraping to document cellular migration across the wound. For the migration assay, 1 × 10^5^ transfected cells were plated in serum-free culture medium in the upper chamber of 24-well Transwell chambers (Corning, Corning, NY, USA), while medium containing 10% fetal bovine serum was added to the lower chamber as a chemoattractant. After the cells were incubated for 48 h, the cells adhering to the lower surface were stained by Crystal Violet, and then counted.

### Alkaline comet assay

Single cell gel electrophoresis was performed using the Comet Assay Kit (4250–050-K; Trevigen, Gaithersburg, MD, USA) according to the manufacturer’s protocol. Briefly, the cells (1 × 10^5^/mL) were mixed with molten low melting point agarose at a ratio of 1:10 (v/v) and layered onto the CometSlide™. The slides were then incubated with the lysis solution at 4 °C for 45 min and with Alkaline Unwinding solution for another 20 min at room temperature. Following an electrophoresis at 21 V for 30 min, excess Neutral Electrophoresis Buffer was drained and the slides were gently immersed in DNA Precipitation Solution (NH_4_OAc, 95% ethanol) for 30 min at room temperature, followed by immersion in 70% ethanol for 30 min at room temperature. The slides were allowed to dry at 37 °C for 10–15 min and were then stained with 4,6-diamidino-2-phenylindole (DAPI; Abcam, Cambridge, UK) at 37 °C for 5 min in the dark and were observed with an Olympus DP71 fluorescence microscope (Olympus, Tokyo, Japan). The tail-moment values of 100 cells were scored using TriTek Comet Score, version 1.5 software (TriTek, Warsaw, Poland).

### Measurement of intracellular reactive oxygen species (ROS) levels

The intracellular ROS levels were evaluated by measuring the dihydroethidium (DHE) (Beyotime, Beijing, China) fluorescence intensity. The cells (1 × 10^5^ cells/well) were washed and harvested in PBS, and then trypsinized, pelleted by centrifugation at 1,500 rpm for 5 min, and resuspended in a serum-free RPMI-1640 medium containing 10 μM DHE for 20 min at 37 °C in the dark. Following the incubation, the cells were subsequently washed and resuspended in 1× PBS. Flow cytometry was performed using a BD Biosciences FACScan II cytometer (BD Biosciences). At least 10,000 cells were collected.

### Immunofluorescence staining

The cells were plated in 6-well plates on coverslips and 1 day later were washed in PBS, fixed in 4% paraformaldehyde for 15 min, and permeabilized for 10 min in 0.2% TritonX-100, and then blocked in 10% normal goat serum for 1 h at room temperature. The cells were then incubated overnight at 4 °C with a primary antibody. The primary antibody was anti-phospho-H2AX (Ser139) (1:500) (Millipore) in Immunol Staining Primary Antibody Dilution Buffer (Beyotime). Visualization was achieved using rhodamine-conjugated secondary antibody (Jackson ImmunoResearch, West Grove, PA, USA). The cells were counterstained with DAPI for nuclear staining. All immunocytochemical markers were observed using a DP71 fluorescence microscope with a 40× objective (Olympus). For each condition, at least 3 coverslips were analyzed. Images from 3 representative high power fields per slide were acquired.

### *In vivo* experiments

Five- to 6-week-old BALB/c nude male mice were purchased from Beijing Vital River Laboratory Animal Technology (Beijing, China). For the tumorigenicity assay, 2 × 10^6^ KYSE30-Tet on-shRECQL4 cells or 2 × 10^6^ TE-1-Tet on-shRECQL4 cells in 0.2 mL of PBS were injected subcutaneously into the right flanks of nude mice (10 mice per group). Before excision, for 3–4 weeks, half the mice were fed with water containing doxycycline (200 μg/mL), and the other half were fed with only water as a negative control^28^. Tumors were measured every 2 days using a caliper, and the volume was estimated using the following formula: volume = length × width^2^/2. All nude mouse experiments were approved by the Institutional Animal Care and Use Committee of Shandong University School of Medicine (No. ECSBMSSDU2019-2-088).

### Statistical analysis

Statistical analysis was performed using SPSS software (Statistical Package for the Social Sciences, version 21.0; SPPS, Chicago, IL, USA). The data were expressed as the mean ± SEM. Differences between two groups were analyzed using the *t*-test. The chi-square test or Fisher’s exact test was used to analyze differences between clinical pathological variables; Kaplan-Meier estimates and log-rank tests were used for survival analyses. A value of *P* < 0.05 was considered statistically significant.

## Results

### RECQL4 is highly expressed in ESCC tissues

**Figure 1:**
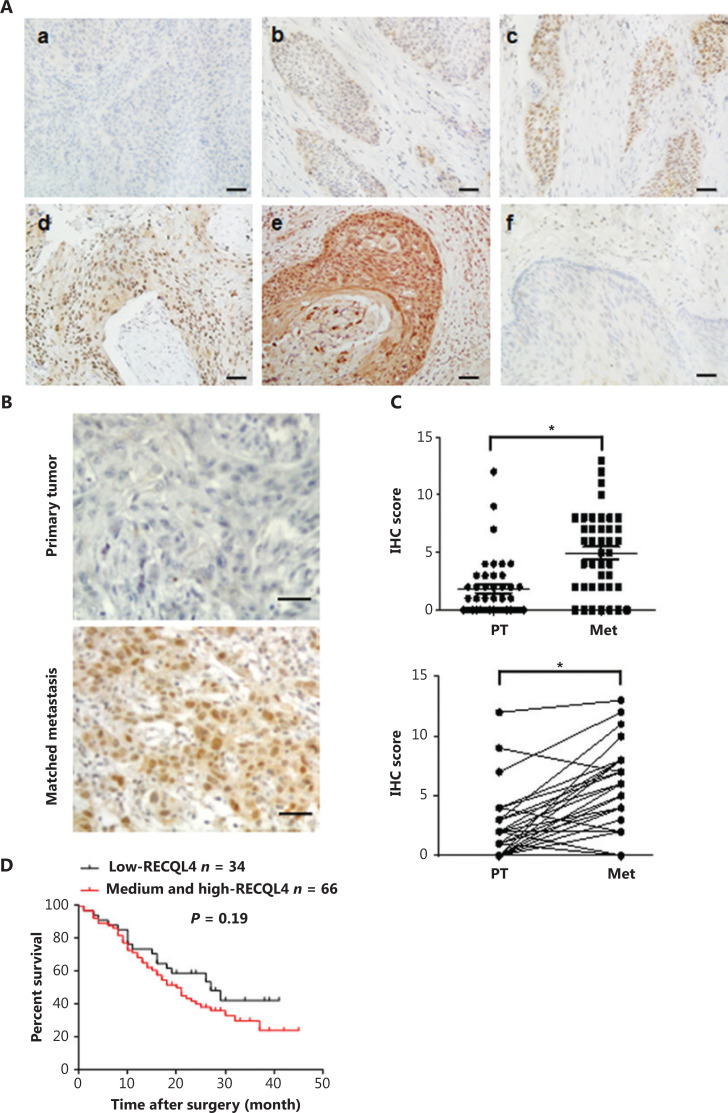
RECQL4 is upregulated in esophageal squamous cell carcinoma (ESCC) and high expression of RECQL4 is correlated with tumorigenesis and metastasis. (A) Immunohistochemical staining of RECQL4 expression in ESCC. Images showing different expression levels and cellular distributions are shown. (a) The absence of RECQL4 staining in ESCC; (b) low levels of RECQL4 in the nuclei of ESCC; (c) intermediate levels of RECQL4 in the nuclei of ESCC; (d) high levels of RECQL4 in the nuclei of ESCC; (e) high levels of RECQL4 in both the cytoplasm and nuclei of ESCC; (f) absence of RECQL4 staining in adjacent non-tumor esophageal tissues. Immunohistochemistry was conducted as described in the Materials and methods. All original images were captured at ×400 magnification; scale bars = 20 μm. (B) Images of immunohistochemistry staining of RECQL4 expression in ESCC cancers and lymph node metastatic carcinoma are shown. All original images were captured at ×400 magnification. Scale bars = 20 μm. (C) A total of 41 pairs of ESCCs and lymph node metastatic carcinomas, and expression levels of RECQL4 were evaluated by immunohistochemistry. (D) Cumulative overall survival curves of 100 ESCC patients with different RECQL4 expressions (high and medium or low). The *P* values were calculated using the log-rank test. *P < 0.05; **P < 0.01; ***P < 0.005.

RECQL4 protein expression was first examined in 197 surgical specimens taken from ESCC patients and the paired adjacent non-tumor tissues using an IHC assay (**Figure 1A**). In the 197 ESCC tissue samples, RECQL4 showed high expression in 44 cases (22.3%), medium expression in 79 cases (40.1%), and low expression in 74 cases (37.6%) (**Table 1**). In contrast, RECQL4 expression was low or absent in adjacent non-tumor tissues (*P* < 0.001, **Table 1**). In specimens exhibiting positive staining of RECQL4, the subcellular distribution varied. Seventy-nine samples showed predominant nuclear staining, 16 samples showed predominant cytoplasmic staining, and 95 samples showed both cytoplasmic and nuclear staining. The above results indicated that RECQL4 was upregulated in ESCC tissues.

**Table 1:** Comparison of RECQL4 expression between tumor tissue and adjacent non-tumor tissue in patients with ESCC

Category	Cases	RECQL4 staining intensity			*P*
		Low, *n*	Medium, *n*	High, n	
Tumor tissue	197	74	79	44	< 0.001
Adjacent non-tumor tissue	55	55	0	0	

IRS score distribution in adjacent non-tumor tissues					
IRS score	0	1	2	3	4
*n* = 55	39	4	0	4	8

IRS score distribution in tumor tissues									
IRS score	0	1	2	3	4	6	8	9	12
*n* = 197	7	15	9	5	38	39	40	5	38

### RECQL4 expression is correlated with clinicopathological features in ESCC patients

We next evaluated the clinical significance of RECQL4 in ESCC. Based on the results of immunohistochemical staining of RECQL4 in ESCC tissues, the association between RECQL4 expression and clinicopathological features was analyzed. As shown in **Table 2**, RECQL4 expression strongly correlated with tumor differentiation (*P* = 0.011), depth of invasion (*P* = 0.033), and lymph node metastasis (*P* = 0.048). However, the expression level of RECQL4 was not significantly associated with sex, age at surgery, tumor size, or tumor stage.

**Table 2:** Relationship between RECQL4 expression and the clinicopathological features of ESCC patients

Characteristics	Cases	RECQL4 staining intensity			*P*
		Low, *n*	Medium, *n*	High, *n*	
Gender					0.056
Male	164	64	67	33	
Female	33	10	12	11	
Age, years					0.062
≥ 60	122	48	48	26	
< 60	75	26	31	18	
Tumor size					0.074
≥ 5 cm	83	33	30	20	
< 5 cm	114	41	49	24	
Tumor differentiation					0.011*
Well	35	18	12	5	
Moderate	119	45	48	26	
Poor	43	11	19	13	
Depth of invasion					0.033*
T1–T2	44	20	15	9	
T3–T4	153	54	64	3	
Lymph node status					0.048*
Negative	71	34	27	10	
Positive	126	41	55	30	
Tumor stage					0.144
I–II	80	38	24	16	
III–IV	117	36	55	28	

*P < 0.05.

We further measured the expression levels of RECQL4 in lymph node metastases and primary tumors from 41 patients with lymph node metastatic ESCCs using immunohistochemistry, after which we rated the amount of RECQL4 staining by using an immunoreactive score (IRS) (**Figures 1B, 1C**). We found that RECQL4 expression was higher in lymph node metastases samples than in the primary tumors (*P* < 0.05). Kaplan-Meier survival analyses showed that ESCC patients with higher RECQL4 expression had no significantly shorter overall survival time than those patients with lower RECQL4 expression (*P* = 0.19) (**Figure 1D**). Taken together, the results showed that the expression of RECQL4 was upregulated during the clinical progression of ESCC, indicating that the expression of RECQL4 may have induced the progression of ESCC cancer.

### Silencing of RECQL4 inhibits ESCC cell proliferation

**Figure 2:**
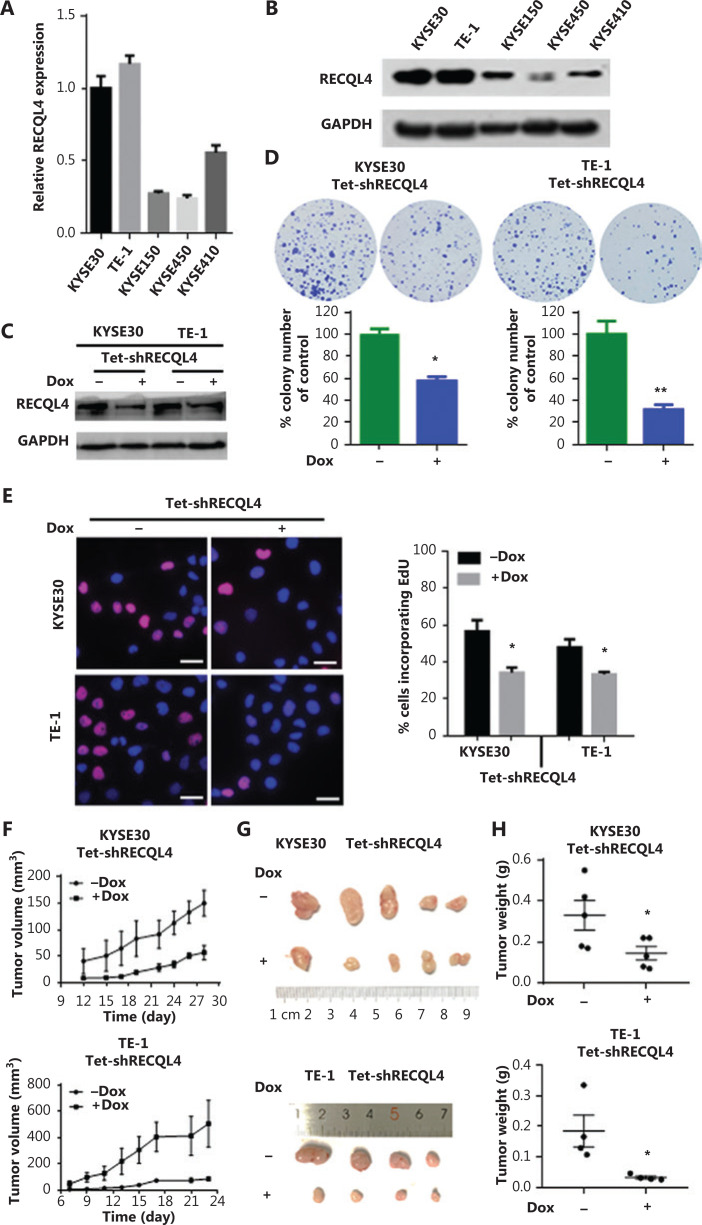
Depletion of RECQL4 inhibits the proliferation of esophageal squamous cell carcinoma (ESCC) cells. Expression analyses of RECQL4 mRNA and protein in 5 ESCC cell lines by (A) qRT-PCR and (B) Western blot. Each bar represents the mean ± SD of 3 replicates. (C) Tet-on inducible shRNA lentiviral vector [LV3 (H1/GFP&Puro)] and tet-on inducible RECQL4 shRNA lentiviral vectors were transfected into 2 higher RECQL4 expression cell lines (KYSE30 and TE-1 cells). Stably transfected cells were obtained by selection with puromycin. Cells were treated with doxycycline (Dox) (500 ng/mL) for 24 h to repress the expression of RECQL4. The cells without Dox treatment were used as controls. The RECQL4 knockout efficiency was examined by Western blot. (D) The clone formation assay in stable RECQL4 knockdown cell lines (KYSE30 and TE-1 cells) with Dox treatment (+Dox) and controls (–Dox). (E) The EdU incorporation assay in stable RECQL4 knockdown cell lines (KYSE30 and TE-1 cells) with Dox treatment (+Dox) and controls (–Dox). EdU incorporation was measured by immunofluorescence staining of EdU (red) and 4′,6-diamidino-2-phenylindole (blue) under the same microscopic magnification (×200). Scale bar, 50 μm. Left, representative EdU incorporation; right, quantitation of EdU incorporation. The number of EdU-positive cells per 200 nucleated cells was determined. *P < 0.05 *vs.* the control. (F–H) RECQL4 inhibited the proliferation of ESCC *in vivo*. (F) A plot of tumor volume over time. Tumor xenograft volumes in Tet-on inducible shRNA-RECQL4-treated nude mice were smaller than those in the scramble group. Tumor graft mass was measured every 3 days, and the volume was calculated using the following formula: volume = length × width^2^/2. (G and H) The dissected xenografts were photographed and weighed at the endpoint. *P < 0.05 *vs.* the control.

To investigate whether RECQL4 played a role in the biological behavior of ESCC cells, we first determined the mRNA and protein levels of RECQL4 in 5 ESCC cell lines, KYSE30, TE-1, KYSE150, KYSE450, and KYSE410, using qRT-PCR and Western blot assays. RECQL4 expression was higher in KYSE30 and TE-1 cells, and lower in the other three cell lines (**Figure 2A, 2B**). We then silenced RECQL4 expression in ESCC cells using a lentiviral vector carrying a specific Tetracycline-inducible (Tet-on) shRNA. A considerable reduction of RECQL4 was achieved in KYSE30 shRNA-RECQL4 and TE-1 shRNA-RECQL4 cells in the presence of doxycycline (+Dox) (**Figure 2C**). The colony formation and EdU incorporation assays demonstrated that RECQL4 knockdown inhibited colony formation (**Figure 2D**) and the proliferation (**Figure 2E**) of ESCC cells. In addition, we established a subcutaneous xenograft model to determine the role of RECQL4 in tumor growth *in vivo*. KYSE30 shRNA-RECQL4 and TE-1 shRNA-RECQL4 cells with doxycycline-inducible RECQL4 knockdown were implanted subcutaneously into the flanks of athymic male nude mice (*n* = 5 per group). RECQL4 knockdown was induced by 1.2 mg/mL of doxycycline administered in 5% sucrose-containing drinking water. The growth of tumors formed by RECQL4 knockdown cells was slower than that of the control cells at all time points analyzed (**Figure 2F**). The average size and weight of tumors formed by RECQL4 knockdown cancer cells at the final experimental endpoint was significantly reduced (**Figure 2G, 2H**). Together, these results indicated that RECQL4 was required for ESCC cell proliferation, both *in vitro* and *in vivo*.

### Overexpression of RECQL4 promotes ESCC cell proliferation

**Figure 3:**
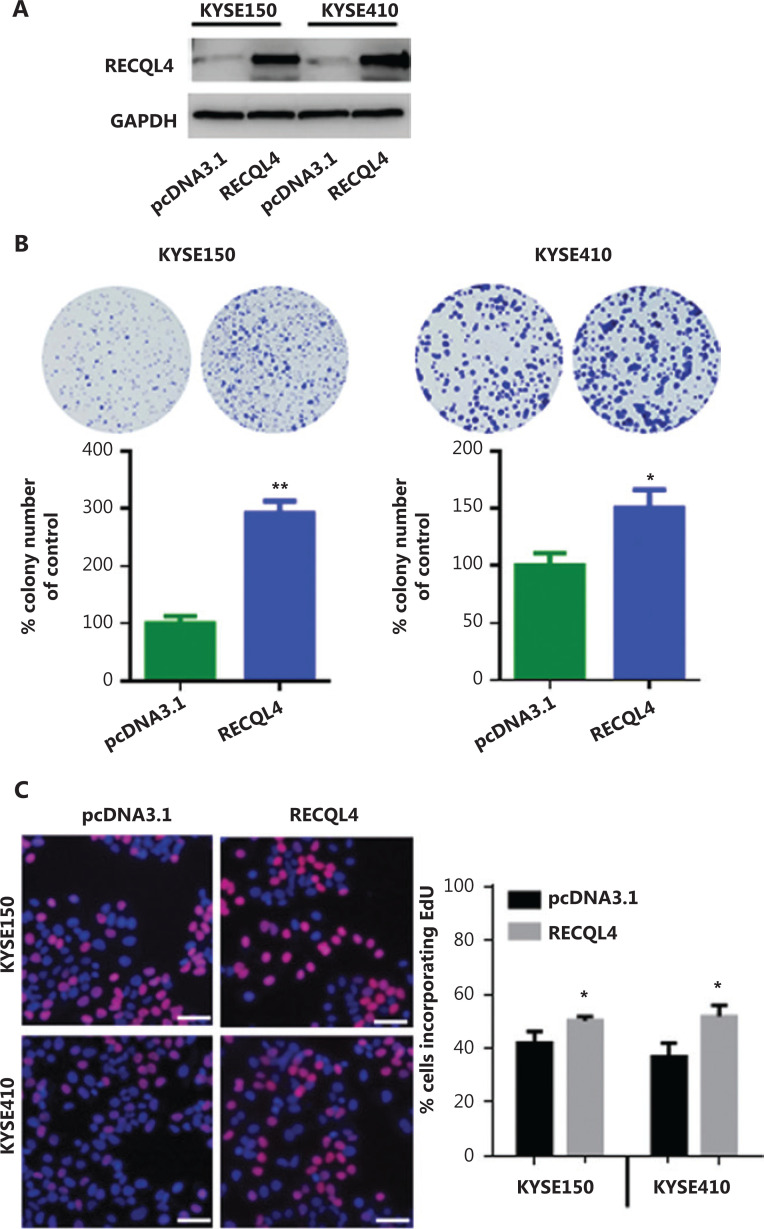
RECQL4 overexpression promotes the proliferation of esophageal squamous cell carcinoma (ESCC) cells. (A) The RECQL4 overexpression efficiency in RECQL4-overexpressing ESCC cell lines (RECQL4) and control cells (pcDNA3.1) assessed by Western blot. (B) The clone formation assay in RECQL4-overexpressing ESCC cell lines (KYSE150 and KYSE410 cells) and controls. (C) EdU incorporation assay in RECQL4-overexpressing ESCC cell lines (KYSE150 and KYSE410 cells) and controls. EdU incorporation was measured by immunofluorescence staining of EdU (red) and 4′,6-diamidino-2-phenylindole (blue) under the same microscopic magnification (×200). Scale bar, 50 μm. The number of EdU-positive cells per 200 nucleated cells was determined. *P < 0.05 *vs.* the control. **P < 0.01 *vs*. the control.

To assess the effect of increased expression of RECQL4 on ESCC cells, we next established stable RECQL4-overexpressing ESCC cells, KYSE150-RECQL4, and KYSE410-RECQL4. ESCC cells transduced with empty pcDNA3.1 vectors were used as negative controls. Western blot analysis confirmed the significantly increased RECQL4 expression in KYSE150-RECQL4 and KYSE410-RECQL4 cells when compared to control cells (**Figure 3A**). Both the KYSE150-RECQL4 and KYSE410-RECQL4 cells displayed a significant proliferation advantage over the respective control cells, as revealed by the colony formation and EdU incorporation assays (*P* < 0.05; **Figures 3B, 3C**), confirming that RECQL4 promoted cell proliferation.

### Depletion of RECQL4 induces G1/G0 cell cycle arrest and cellular senescence

**Figure 4:**
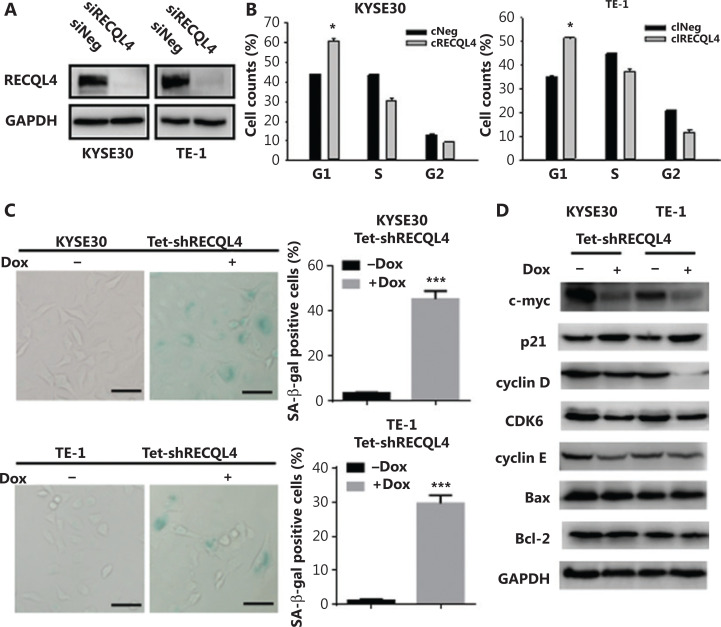
The loss of RECQL4 induces cell cycle arrest and cellular senescence. (A) Depletion of RECQL4 by siRNA. RECQL4 protein levels were measured by Western blot. KYSE30 and TE-1 cells were transfected with siRNA duplexes (200 nM) specific to RECQL4 or negative oligo in serum-free medium for 4 h, then replaced with complete medium for 24 h. Whole cell extracts were collected for Western blot analysis using RECQL4 antibodies. (B) Cell cycle distributions in RECQL4 knockdown cell lines (KYSE30 and TE-1 cells) and controls were determined by flow cytometry. (C) Cellular senescence was examined by SA-β-gal staining. Microscopic magnification (×200), Scale bar: 50 μm. (D) The protein levels of c-myc, p21, cyclin D, CDK6, cyclin E, Bax, and Bcl-2 were determined by Western blot in stable Tet-on inducible RECQL4 knockdown cell lines (KYSE30 and TE-1 cells) (+Dox) and controls (–Dox). Experiments were independently repeated 3 times. All data indicate the mean ± SD. *P < 0.05; **P < 0.01; ***P < 0.001.

We next identified the potential mechanism underlying the inhibitory effect of RECQL4 depletion on cell proliferation. For this purpose, cells in exponential growth phase (KYSE30 and TE-1) were transfected with 200 nmol/L of either control siRNA or RECQL4-specific siRNA (**Figure 4A**). Silencing of RECQL4 caused more cells to arrest in the G1 phase and fewer cells to enter S and G2 phases, when examined at 72 h after transfection, indicating G1/G0 arrest (**Figure 4B**).

Cellular senescence, a state of irreversible cell-cycle arrest, can cause loss of proliferative capacity. We therefore measured SA-β-gal activity, as a marker of senescence, in RECQL4 knockdown and control cells. Approximately 45% of KYSE30 RECQL4-depleted cells and 30% of TE-1 RECQL4-depleted cells showed positive SA-β-gal staining (**Figure 4C**). The expression of p21, an effector molecule of G1 arrest and cellular senescence, was also increased significantly (**Figure 4D**), which is consistent with previous studies^29^. However, knockdown of RECQL4 had no significant impact on apoptosis in both KYSE30 and TE-1 cells (**Supplementary Figures S1 and 4D**).

To further investigate how RECQL4 regulates G1/S phase transition and cellular senescence, we determined the expression of other related proteins. As shown in **Figure 4D**, depletion of RECQL4 led to decreased expression of c-myc, cyclin D, CDK6, and cyclin E. Overall, these results showed that the inhibitory effects of RECQL4 depletion on the proliferation of ESCC cells might be mediated by p21 upregulation and the downregulation of mitogenic genes.

### RECQL4 promotes the epithelial-mesenchymal transition (EMT) in ESCC cells

**Figure 5:**
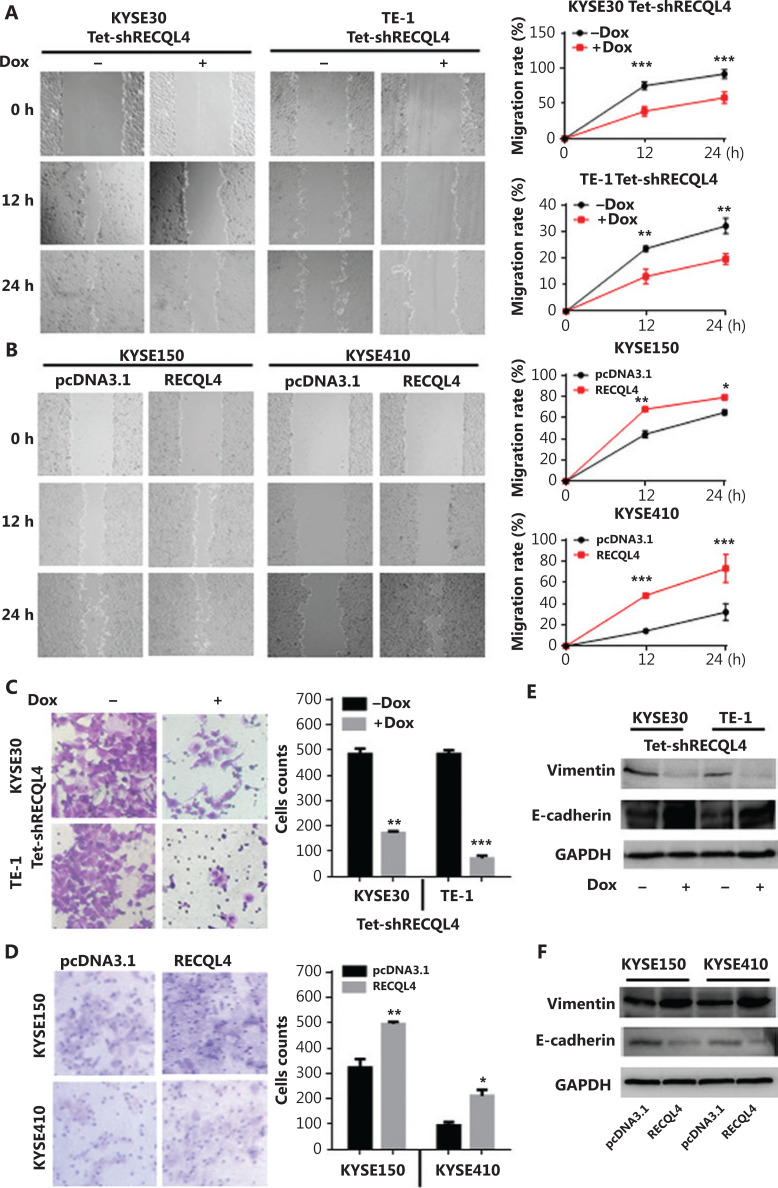
RECQL4 promotes the epithelial-mesenchymal transition (EMT) in esophageal squamous cell carcinoma (ESCC) cells. (A) Cell migration capacities in stable Tet-on inducible RECQL4 knockdown cell lines (KYSE30 and TE-1 cells) (+Dox) and controls (–Dox) were determined using the scratch-wound assay. (B) Cell migration capacities in RECQL4-overexpressing ESCC cell lines (KYSE150 and KYSE410 cells) and controls were determined using the scratch-wound assay. Photographs were taken at 0, 12, and 24 h after scratching. We calculated the migration rate using the following formula: [1 – (current denuded zone area/initial denuded zone area)] × 100. (C) Cell migration capacities in stable Tet-on inducible RECQL4 knockdown cell lines (KYSE30 and TE-1 cells) (+Dox) and controls (–Dox) were determined using the Transwell assay. (D) Cell migration capacities in RECQL4-overexpressing ESCC cell lines (KYSE150 and KYSE410 cells) and controls were determined using the Transwell assay. (E) The protein levels of EMT markers (E-cadherin and Vimentin) were detected by Western blot in stable Tet-on inducible RECQL4 knockdown cell lines (KYSE30 and TE-1 cells) (+Dox) and controls (–Dox). (F) The protein levels of EMT markers (E-cadherin and Vimentin) were detected by Western blot in RECQL4-overexpressing ESCC cell lines (KYSE150 and KYSE410 cells) and controls. Experiments were independently repeated 3 times. All data indicate the mean ± SD. *P < 0.05; **P < 0.01; ***P < 0.001.

The data from clinical ESCC specimens showed that RECQL4 overexpression was positively correlated with lymph node metastasis. The EMT is a central mechanism contributing to invasion and metastasis of various cancers^30,31^. To test whether RECQL4 contributed to the EMT, we used scratch-wound and Transwell assays to determine the role of RECQL4 in ESCC cell migration. Cell-free areas in the RECQL4 shRNA groups were larger than those in the control groups at 12 h and 24 h after scratches were made (**Figure 5A**). Conversely, cell-free areas in the RECQL4 overexpression groups were smaller than those in the control groups (**Figure 5B**). The Transwell assay showed that RECQL4 depletion inhibited cell migration (**Figure 5C**), while RECQL4 upregulation enhanced cell migration (**Figure 5D**). We next measured the protein levels of EMT markers by Western blot analysis. As shown in **Figure 5E**, the expression levels of epithelial markers such as E-cadherin were increased, while the expression levels of mesenchymal markers such as vimentin were reduced in RECQL4-knockdown cells. Opposite changes were observed in ESCC cells with RECQL4 overexpression (**Figure 5F**). Taken together, these results suggested that RECQL4 promoted the EMT-mediated metastasis in ESCC.

### Stable RECQL4 knockdown increases ROS production, spontaneous DNA strand break accumulation, and impairs DNA damage response in ESCC cells

**Figure 6:**
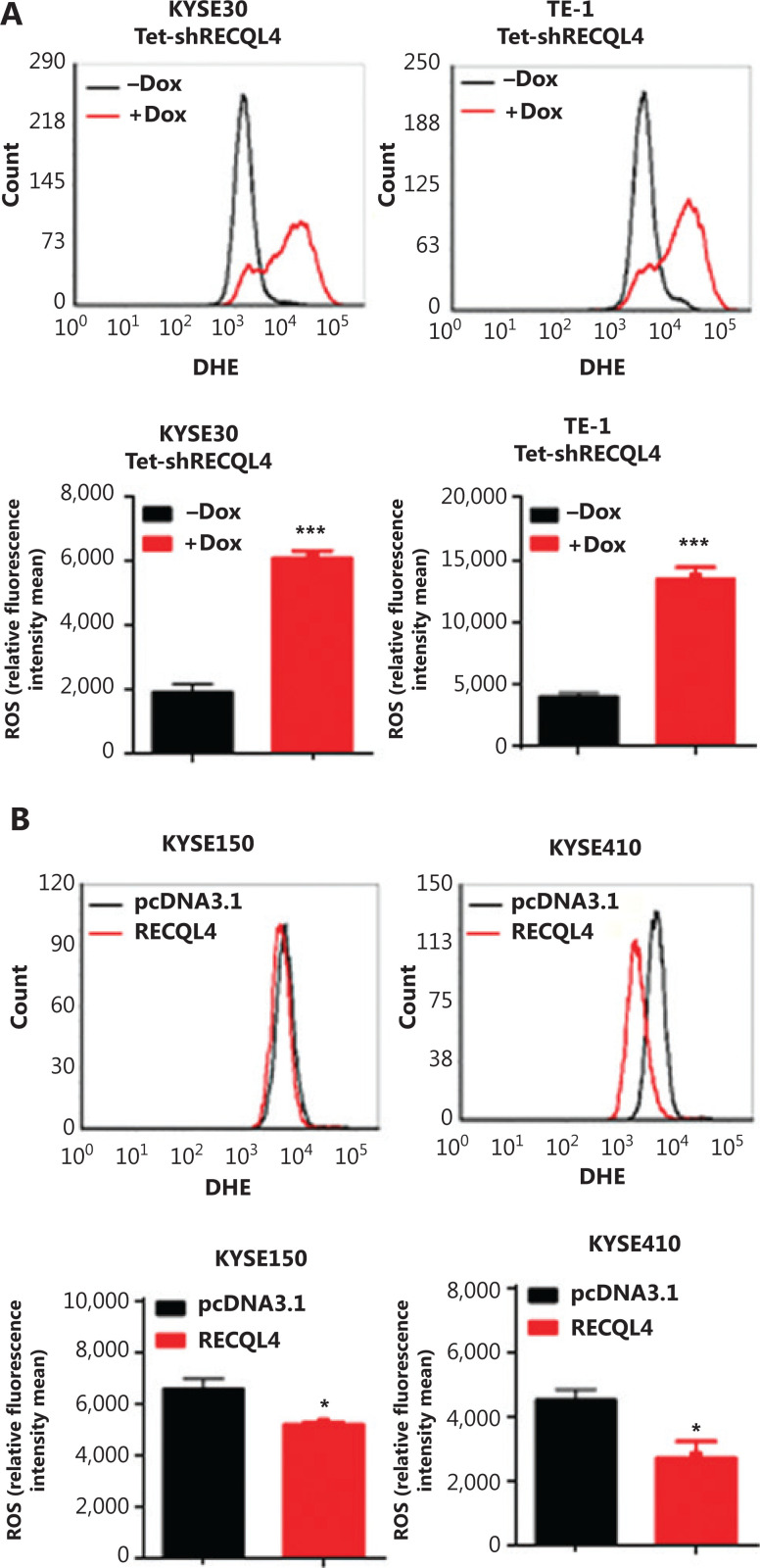
RECQL4 regulates reactive oxygen species (ROS) production in esophageal squamous cell carcinoma (ESCC) cells. ROS generation was measured using the oxidation-sensitive fluorescent probe (DHE) in stable Tet-on inducible RECQL4 knockdown cell lines (KYSE30 and TE-1 cells) (+Dox) and controls (-Dox) (A), and RECQL4-overexpressing ESCC cell lines (KYSE150 and KYSE410 cells) and controls (B) by flow cytometry. Means and SDs of 3 repeats are shown at the bottom. *P < 0.05; ***P < 0.001.

The induction of senescence and cell cycle arrest are often attributed to high levels of ROS and spontaneous DNA damage. To test whether RECQL4 depletion caused oxidative stress, we measured the levels of ROS in KYSE30 shRNA-RECQL4, and TE-1 shRNA-RECQL4 cells using DHE as a probe. As shown in **Figure 6A**, RECQL4 knockdown for 72 h led to an increase in the generation of ROS in both cell lines tested. In contrast, RECQL4 overexpression decreased ROS levels (**Figure 6B**). These results indicated that RECQL4 contributed to the maintenance of redox homeostasis in ESCC cells.

**Figure 7:**
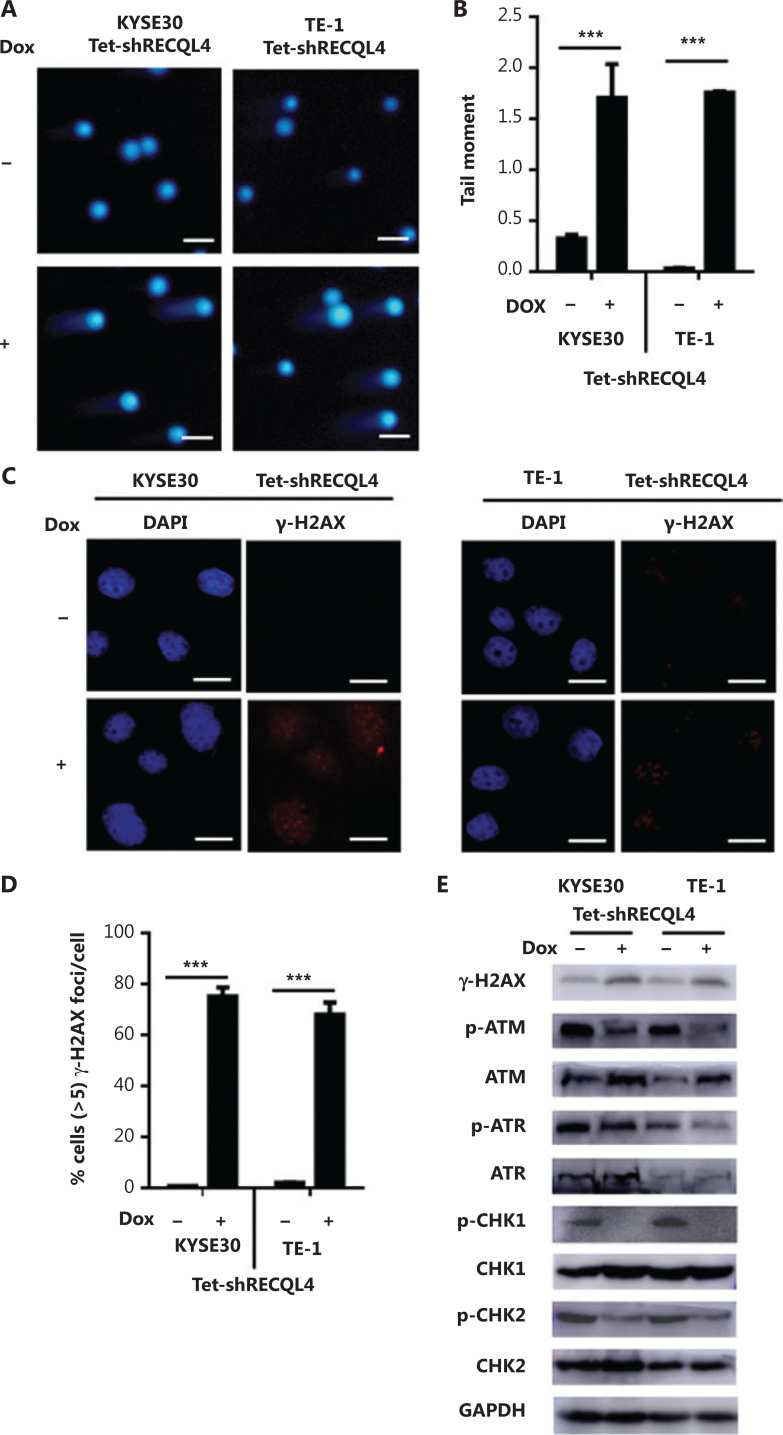
Depletion of RECQL4 causes DNA damage, but impairs the DNA damage response in esophageal squamous cell carcinoma (ESCC) cells. (A) Stable Tet-on inducible RECQL4 knockdown cell lines (KYSE30 and TE-1 cells) (+Dox) and controls (–Dox) were analyzed using the alkaline comet assay, and the tail moment was calculated as the percentage DNA in the tail multiplied by the tail length, with a microscopic magnification at ×200. Scale bar: 100 μm. (B) Statistical analysis of the data derived from (A). ***P < 0.001. (C) Immunofluorescence staining of γ-H2AX in stable Tet-on inducible RECQL4 knockdown cell lines (KYSE30 and TE-1 cells) (+Dox) and controls (–Dox). Microscopic magnification (×400), Scale bar: 20 μm. (D) Distribution of cells with at least 5 γ-H2AX foci. For each group 500 cells were counted. Shown are the averages and SD of 3 repeats. ***P < 0.001. (E) Western blot analysis of γ-H2AX, p-ATM, ATM, p-ATR, ATR, p-CHK1, CHK1, p-CHK2, and CHK2 protein levels in stable Tet-on inducible RECQL4 knockdown cell lines (KYSE30 and TE-1 cells) (+Dox) and controls (–Dox).

We next measured the levels of DNA damage in KYSE30 shRNA-RECQL4 and TE-1 shRNA-RECQL4 cells using an alkaline comet assay. As shown in **Figure 7A**, RECQL4 knockdown for 72 h led to an increase in the generation of strand breaks in both cell lines. In addition, the phosphorylation of H2AX at Ser139, or γ-H2AX, a marker of DSBs, was also elevated in KYSE30 shRNA-RECQL4 and TE-1 shRNA-RECQL4 cells treated with Dox for 72 h, as indicated by Western blot and immunofluorescence analyses (**Figures 7B–D**). Together, these results indicated that depletion of RECQL4 resulted in increased accumulation of DNA damage in ESCC cells.

Cellular responses to DNA damage are coordinated primarily by two distinct kinase signaling cascades, the ATM-CHK2 and ATR-CHK1 pathways. The activation of ATM/ATR and their downstream effectors, CHK1 and CHK2, was therefore examined in RECQL4-depleted cells. Surprisingly, RECQL4 depletion resulted in a significant reduction in the phosphorylation of ATM and ATR, markers for their activation (**Figure 7E**). The depletion of RECQL4 also resulted in the decrease in the phosphorylation of CHK1 and CHK2, suggesting that the activation of the ATM/ATR-dependent checkpoint pathway was impaired in RECQL4-depleted cells.

### RECQL4 is required for sustaining pro-survival NF-*κ*B, PI3K/AKT, and MAPK signaling pathways

**Figure 8:**
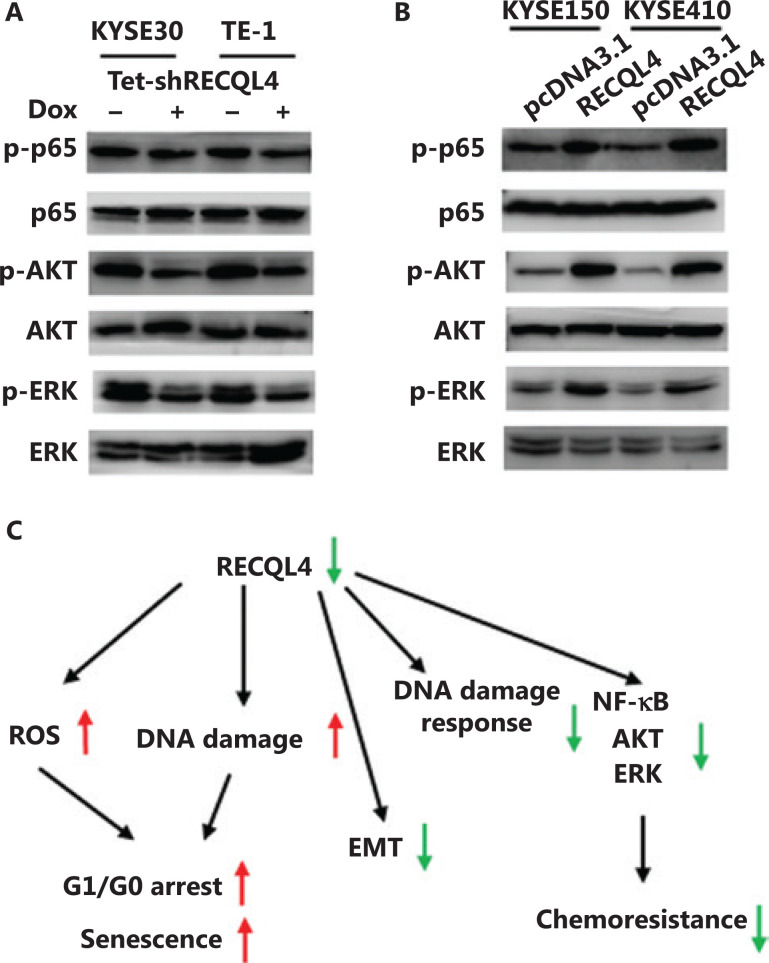
RECQL4 positively regulates NF-κB, AKT, and ERK signaling pathways. (A) The protein levels of p-65, p65, p-AKT, AKT, p-ERK, and ERK were determined by Western blot in stable Tet-on inducible RECQL4 knockdown cell lines (KYSE30 and TE-1 cells) (+Dox) and controls (–Dox). (B) The protein levels of p-65, p65, p-AKT, AKT, p-ERK, and ERK were determined by Western blot analysis in RECQL4-overexpressing ESCC cell lines (KYSE150 and KYSE410 cells) and controls. Experiments were independently repeated 3 times. (C) A schematic model for the function and mechanistic pathways of RECQL4 in esophageal squamous cell carcinomas.

We further investigated the modulation of several oncogenic pathways by functional status of RECQL4. Given that EMT-related genes are downstream targets of NF-κB signaling, and ATM is the upstream kinase of the NF-kB pathway^32,33^, we hypothesized that the impaired ATM activation due to RECQL4 depletion may lead to decreased phosphorylation of p65, a NF-κB subunit. Using Western blot analysis, we found that the levels of p65 phosphorylation were decreased in both KYSE30 and TE-1 cells when RECQL4 was depleted (**Figure 8B**). The opposite changes were observed in ESCC cells overexpressing RECQL4 (**Figure 8B**). The depletion of RECQL4 also caused significant decreases in the phosphorylation levels of AKT and ERK1/2, when compared with the control group (**Figure 8A**). In contrast, RECQL4 overexpression in ESCC cells led to augmented phosphorylation of AKT and ERK1/2 (**Figure 8B**). Thus, RECQL4 positively regulated several pro-survival pathways in ESCC cells.

## Discussion

Although genomic analysis of ESCC has identified several key pathways that drive the pathogenesis of ESCC, the molecular mechanisms responsible for ESCC progression, especially the functions of the proteins involved in DNA metabolism, remain to be fully elucidated. This report is the first investigation of the function of RECQL4 in ESCCs. We found that RECQL4 protein expression was significantly higher in both primary tumors and lymph node metastases than in normal esophageal epithelium. In primary tumors, high RECQL4 expression was positively associated with tumor differentiation, depth of invasion, and lymph node metastasis. By employing ESCC cell lines in which RECQL4 was depleted or ectopically overexpressed, we showed that RECQL4 was required for cell proliferation, resistance to cellular senescence, and migration. Mechanistically, RECQL4 depletion led to increased ROS production and DNA damage accumulation.

Overexpression of RECQL4 due to gene amplification has been reported to be associated with tumor progression of many human malignant cancers^19–24^. For example, in clinical tissue samples of breast cancer, high levels of RECQL4 protein were significantly associated with aggressive tumor behavior, including lymph node positivity, larger tumor size, HER2 overexpression, ER-negativity, triple-negative phenotypes, and poor survival^22^, and shRNA-mediated RECQL4 suppression in MDA-MB453 breast cancer cells significantly inhibited *in vitro* clonogenic survival and *in vivo* tumorigenicity^24^. Higher RECQL4 expression was observed in gastric cancers, and RECQL4 protein levels were associated with poor survival of gastric cancer patients^21,26^. The expression of RECQL4 mRNA in hepatocellular carcinoma (HCC) tissues was significantly higher compared with adjacent normal liver tissues; HCC patients with higher levels of RECQL4 expression exhibited significantly shorter disease-free survival and overall survival times compared with those with low levels of expression^23^. Elevation of RECQL4 level was positively associated with the aggressiveness of prostate cancer both *in vitro* and *in vivo*, implying that RECQL4 plays critical roles in prostate-cancer carcinogenesis and is a valuable biomarker for this cancer^29^. Most notably, RECQL4 knockdown in metastatic prostate cancer cells drastically reduced their cell invasiveness *in vitro* and tumorigenicity *in vivo*^20^. Similarly, our results in the present study indicated that RECQL4 possessed oncogenic properties in ESCCs. RECQL4 appeared to be critical for maintaining the redox homeostasis and genomic integrity in ESCC. Increased oxidative stress and DNA damage caused by RECQL4 depletion could drive cellular senescence and thereby inhibit tumor progression.

Unlike other human RECQ helicases that are only located in the nucleus, RECQL4 is found both in the nucleus and the cytoplasm, and contributes to the integrity of both the nuclear and mitochondrial genomes^22,34–37^. We observed that RECQL4 was localized in the cytoplasm and/or nucleus. It is possible that RECQL4 may function in both nuclei and mitochondria to exert its oncogenic effects. Future studies may reveal whether the increased ROS caused by RECQL4 depletion is mediated by mitochondrial dysfunction.

It is well-known that DNA damage checkpoints employ damage sensor proteins, such as ATM and ATR, to detect DNA damage and to initiate signal transduction cascades that comprise CHK1 and CHK2 kinases^38–40^. The signal transducers activate NF-κB, AKT, and ERK signaling pathways, which are usually hyperactive in multiple cancers, including ESCC, and promotes tumor growth, the EMT and metastasis^41–43^. When the activations of ATM, ATR, CHK1, CHK2, NF-κB, AKT, and ERK were analyzed by Western blot analyses, we surprisingly found that knockdown of RECQL4 significantly reduced the phosphorylation of all these kinases. Furthermore, a recent study reported that the helicase activity of RECQL4 played an important role in the activation of ATM-dependent checkpoint pathway against DNA double strand breaks in human cells^44^. Future studies may therefore reveal how RECQL4 participates in the DNA damage response.

## Conclusions

We showed that RECQL4 was remarkably overexpressed in ESCC tissues, especially in metastases, and was positively correlated with poor tumor differentiation, lymph node metastasis, and advanced stage. RECQL4 was absolutely required for cell proliferation and the EMT. Its contribution to the maintenance of ESCC malignancy may involve its regulation of ROS formation and the DNA damage response. RECQL4 may therefore represent a potential biomarker and a therapeutic target in ESCC.
