# Supplementary material for: RECQL4 regulates DNA damage response and redox homeostasis in esophageal cancer
Source: Cancer Biol Med. 2021 Feb 15;18(1):120–38. doi: 10.20892/j.issn.2095-3941.2020.0105 (PMC7877169; doi:10.20892/j.issn.2095-3941.2020.0105)
Supplement: Supplementary file 1 [file cbm-18-120-s001.pdf]

Supplementary material

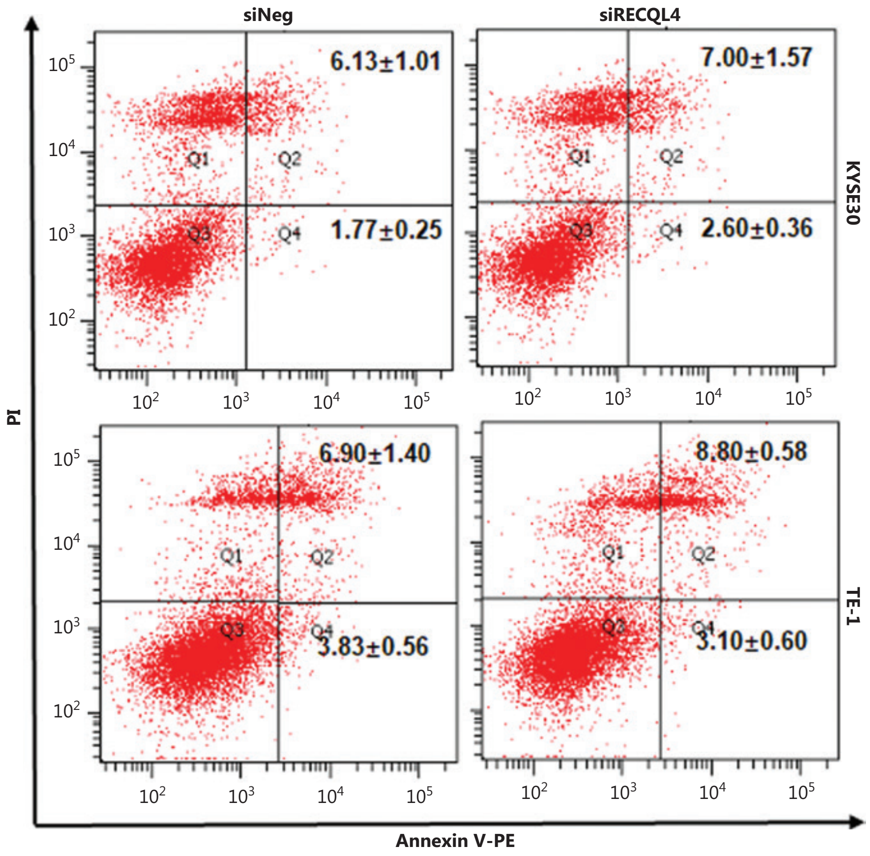

Figure S1 Apoptosis in RECQL4 knockdown cell lines (KYSE30 and TE-1 cells) and controls was determined by flow cytometry. KYSE30 and TE-1 cells were transfected with siRNA duplexes (200 nM) specific to RECQL4 or negative oligo in serum-free medium for 4 h, then replaced with complete medium for 24 h. The percentages of annexin V- or/and propidium-positive cells are indicated.
